# Multi-level examination of correlates of active transportation to school among youth living within 1 mile of their school

**DOI:** 10.1186/1479-5868-9-124

**Published:** 2012-10-16

**Authors:** Kathleen M Gropp, William Pickett, Ian Janssen

**Affiliations:** 1 Department of Community Health and Epidemiology, Queen’s University, Kingston, ON, Canada; 2 Department of Emergency Medicine, Queen’s University, Kingston, ON, Canada; 3 School of Kinesiology and Health Studies, Queen’s University, Kingston, ON, K7L 3N6, Canada

**Keywords:** Active transportation, Adolescent, School, Neighborhood

## Abstract

**Background:**

Active transportation to school is a method by which youth can build physical activity into their daily routines. We examined correlates of active transportation to school at both individual- (characteristics of the individual and family) and area- (school and neighborhood) levels amongst youth living within 1 mile (1.6 km) of their school.

**Methods:**

Using the 2009/10 Canadian Health Behaviour in School-Aged Children (HBSC) survey, we selected records of students (n = 3 997) from 161 schools that resided in an urban setting and lived within 1 mile from their school. Student records were compiled from: (1) individual-level HBSC student questionnaires; (2) area-level administrator (school) questionnaires; and (3) area-level geographic information system data sources. The outcome, active transportation to school, was determined via a questionnaire item describing the method of transportation that individual students normally use to get to school. Analyses focused on factors at multiple levels that potentially contribute to student decisions to engage in active transportation. Multi-level logistic regression analyses were employed.

**Results:**

Approximately 18% of the variance in active transportation was accounted for at the area-level. Several individual and family characteristics were associated with engagement in active transportation to school including female gender (RR vs. males = 0.86, 95% CI: 0.80-0.91), having ≥2 cars in the household (RR vs. no cars = 0.87, 0.74-0.97), and family socioeconomic status (RR for ‘not well off’ vs. ‘very well off’ = 1.14, 1.01-1.26). Neighborhood characteristics most strongly related to active transportation were: the length of roads in the 1 km buffer (RR in quartile 4 vs. quartile 1 = 1.23, 1.00-1.42), the amount of litter in the neighborhood (RR for ‘major problem’ vs. ‘no problem’ = 1.47, 1.16-1.57), and relatively hot climates (RR in quartile 4 vs. quartile 1 = 1.33 CI, 1.05-1.53).

**Conclusion:**

Engagement in active transportation to school was related to multiple factors at multiple levels. We identified gender, perception of residential neighborhood safety, the percentage of streets with sidewalks, and the total length of roads as the most important correlates of active transportation to school.

## Introduction

Active transportation is the engagement in physical activity specifically for travel and includes methods such as walking and bicycling [1]. Active transportation is one means by which children and youth can incorporate physical activity into their daily routines. Indeed, children and youth who walk or bicycle to school have higher overall physical activity [2] and cardiorespiratory fitness levels [3] and a healthier body composition [3]. Unfortunately, the proportion of children and youth who engage in active transportation to school has decreased by 8-10% in Canada over the last two decades [4] and by 25% in the United States over the last four decades [5], which is of obvious concern to public health. Evidence about the various factors that lead to decisions to engage in active transportation to school is fundamental to the development of effective health promotion strategies.

Correlates of active transportation to school exist at multiple levels, including characteristics of individual students and their families (individual-level) and characteristics of the students’ schools and their neighborhoods (area-level). Individual and family characteristics that may be relevant include living in close proximity to school [6-9], male gender [6,9-11], ethnicities other than Caucasian [6,12,13], low family socio-economic status [14,15], and a non-traditional family structure [14,15]. While there is little information about the influence of schools and school policies on active transportation to school, one school-based active transportation study found that students attending private school were 40% less likely to engage in regular active transportation [16]. At the neighborhood-level, students who live in densely populated areas are more likely to engage in active transportation to school [12]. Another relevant neighborhood factor is the presence of sidewalks, which has consistently been shown to be a strong correlate [11,16-19]. Associations between active transportation and the design of road networks are less clear. A recent systematic review reported varying associations for intersection density, block length, and route directness [20]. Despite the interest in characteristics of the neighborhood, certain variables have not been examined, including neighborhood aesthetics (e.g., litter, condition of houses and buildings) and safety features (e.g., speed limits of roads surrounding the school).

Although the correlates of active transportation to school occur at multiple levels, the vast majority of existing studies on this topic have not simultaneously considered multiple factors at the various levels [7-9,11,13,18,21-25]. The few multi-level studies that exist have been conducted within small geographic areas [8,9,11,17,22,26], which limits their generalizability. Multi-level research that examines a multitude of potential correlates could help illustrate the complexity behind decisions that govern whether or not youth travel to school in an active or passive manner. Furthermore, such research would help identify the strongest correlates, which in the short-term may be identified for more focused study, and in the long-term may be addressed via preventive interventions.

We conducted a national analysis of possible individual-level and area-level correlates of active transportation to school among urban Canadian youth aged 11–15 years who lived within 1 mile (1.6 km) of their school. Our goal was to identify major factors at multiple levels that govern decisions to engage in active transportation amongst youth not eligible for bussing and who live within a reasonable walking or biking distance from school. This study was exploratory and no a priori hypotheses were assumed, although our choices of variables for study were governed by existing literature.

## Methods

### Overview of study design and measures

The basis for this study was the 2009/10 Canadian Health Behaviour in School-Aged Children (HBSC) Survey. HBSC is a general health survey of grades 6–10 students conducted in affiliation with the World Health Organization. The 2009/10 Canadian HBSC, or the 6^th^ Canadian cycle, consisted of three main components: (1) a questionnaire completed by students that asked about their health behaviors (such as active transportation), lifestyle factors, and demographics, (2) an administrator questionnaire distributed to each school principal that inquired about school demographics, policy, infrastructure, and about the school neighborhood setting, and (3) geographic information systems (GIS) measures of built and social features in the school neighborhoods that were later linked with the HBSC data.

### Participants

In Canada, the HBSC survey follows a systematic multi-stage cluster sample where individual students are nested in school classes, which in turn are nested within schools, followed by school boards. This sampling approach adheres to the standard international protocol [27]. In 2009/10 the HBSC survey was administered to 26 078 Canadian students in grades 6–10 from 436 schools in 11 territories and provinces (all jurisdictions with the exception of Prince Edward Island and New Brunswick participated). With respect to human subjects, consent to participate was obtained from school boards, individual schools, parents or guardians (either explicitly or implicitly determined by school board policy), and from individual students. Ethics approval for the Canadian HBSC was granted by the General Research Ethics Board of Queen’s University.

For this study, we only included participating students who attended school in an urban core, as indicated by the school postal codes. An urban core is defined as a large urban area that has a population of at least 50 000 in the urban core in the case of a Census Metropolitan Area, or a population of at least 10 000 in the urban core for a Census Agglomeration”. In Canada, postal codes provide specific indicators of location of residence in urban core settings, but not in rural locations [28].

**Figure 1:**
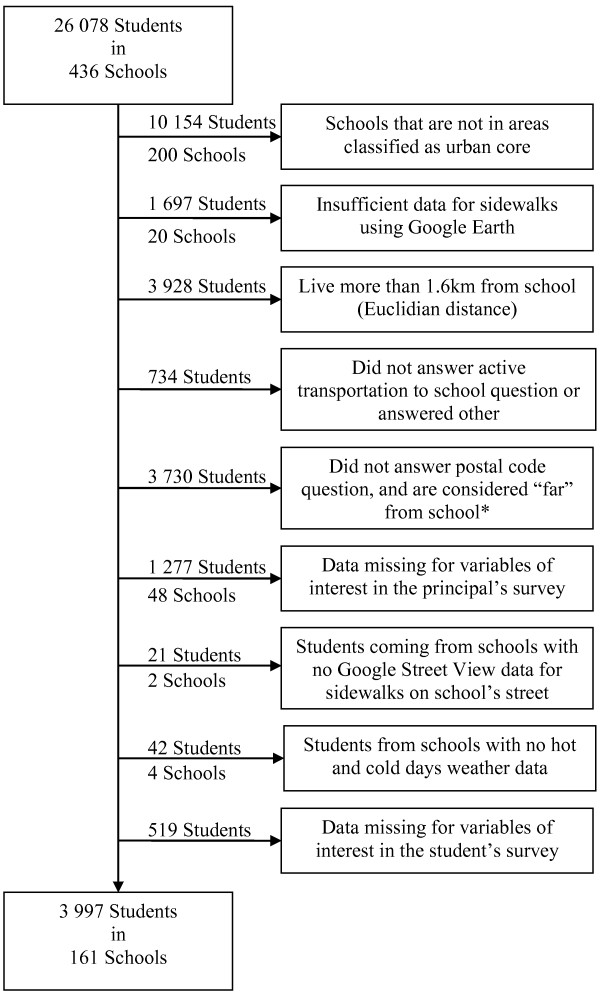
**Exclusion flow-chart.** *Far from school: More than 15 minutes walking, or more than 5 minutes by bike, bus, train, subway, streetcar, ferry/boat, car, motorcycle, moped.

To be included in this study we required that the participants lived within a reasonable walking distance of their school, estimated at 1 mile (1.6 km) or less. Walking distances were conservatively estimated using direct distance from the geographical center of their postal code to the school address. The inclusion criteria limited the study base to students who had a realistic opportunity to regularly engage in active transportation to school. By school board policy, Canadian students who live more than 1 mile from school are typically offered transportation by school bus, although this distance can vary. A large percentage (43%) of urban students did not report their postal code in the HBSC survey, and to increase the study sample size, for these students we used their answers to questions about “travel time to school” and “usual mode of transportation to school” to estimate whether they lived within the 1 mile distance. Students who reported that their travel time to school was 15 minutes or less by walking, or less than 5 minutes for every other mode of transportation (bicycle, car, bus, etc.), were therefore also included. The final sample size of urban youth available for analysis was 3,997 (see Figure 1 for a participant flow diagram).

### Outcome – active transportation to school

The outcome of interest was regular engagement in active transportation to school, either via walking or bicycling. Participants answered the following HBSC survey question: “On a typical day, the MAIN part of your journey TO school is made by…” with the following options: 1) walking; 2) bicycle; 3) bus, train, streetcar, subway, or boat/ferry; 4) car, motorcycle, or moped; 5) other. Responses were grouped dichotomously: those who answered “walking” or “bicycle” were categorized as students who regularly engage in active transportation to school, while those who answered ‘bus, train, streetcar, subway, boat/ferry, car, motorcycle, moped’ were categorized as individuals who do not. Participants who answered ‘other’ to this question were excluded to minimize possible misclassification. Intra-rater reliability analyses for the HBSC active transportation question suggest there is an excellent level of agreement (Cronbach’s alpha ≥0.80) between multiple student reports, including reports examined across seasons [29].

### Possible correlates of active transportation

We constructed a list of possible correlates of active transportation to school based on the evidence in existing literature. We then cross-referenced this list against the HBSC and GIS data available to our research team and examined all of the possible correlates that we could.

#### Individual and family correlates (individual-level data)

Eight items describing potential correlates of the individual participants and their families were obtained via the HBSC student questionnaire: gender (male or female), grade (6–8 and 9–10), ethnicity (four composite categories consisting of: Caucasian only, Caucasian and other, Aboriginal, and other), number of siblings (0, 1, 2 or more), family structure (living with both parents, living with one parent and a step-parent, living with a single parent, and all other living situations), family socio-economic status (SES) as measured by perceived relative affluence (5 categories from “very well off” to “not well off at all”), the number of cars in the household (0, 1, 2 or more), and perceived residential neighborhood safety (where you live, is it safe for children to play outside? (5 categories: “strongly agree” to “strongly disagree”).

#### School correlates (area-level data)

Three items of the school were measured using the HBSC administrator questionnaire. These items focused on school active transportation policies, programs, and infrastructure. A series of questions inquired about whether the school promoted active transportation by: (1) having walk and/or bike to school days or walking school buses; (2) identifying safe routes to walk or bicycle to school; and (3) providing bicycle racks in safe locations. All three of these items were dichotomized as “yes” or “no” for analytical purposes. Schools with administrators who answered “don’t know” to these questions were classified as “no”.

#### Neighborhood correlates (area-level data)

##### Aesthetics

Two items that reflect neighborhood aesthetics were measured using the HBSC administrator questionnaire: (1) presence of litter in the school neighborhood; and (2) vacant or shabby housing in the school neighborhood. Four possible response categories were available, ranging from “no problem” to “major problem”.

Twelve items of the neighborhood were measured with GIS using the CanMap Route Logistics database (DMTI Spatial Inc., Markham, ON) in ArcView version 9.3 software, PCensus for MapPoint software (Tetrad Computer Applications Inc., Vancouver, BC), Google Earth and Google Street View software (Google Inc., Mountain View, CA), and Environment Canada data (National Climate Data and Information Archive). Some of these variables were obtained from a 1-km radius circular buffer surrounding the school, some were obtained at the exact school address, and others were obtained for the municipality where the school was located.

##### Sidewalk measures

The first of the GIS measures consisted of whether there was at least one sidewalk leading directly to the school. This was measured using Google Street View. If the Google Street View image confirmed that there was a sidewalk on either side of the street on which the school was located, this variable was categorized as “yes”, otherwise it was categorized as a “no”. The use of Google Street View as an alternative to physical audit has been validated, and measures (such as the presence of recreational buildings and parks) have produced observed agreement correlations of 0.92 and 0.95, respectively [30].

The percentage of roads with sidewalks in the 1 km (0.62 miles) buffer surrounding each school was obtained from Google Earth and ArcGIS, as previously described in detail [31]. The length of roads with a sidewalk (on either side) was gathered and divided by the total length of roads. This variable was then categorized into quartiles. This was done by first calculating the distance of roads in the buffer using the CanMap Route Logistics database in ArcGIS. The road network was exported from ArcGIS into Google Earth, and within Google Earth the road segments were superimposed onto the street view images. Road segments that did not have a sidewalk were deleted from the road network within Google Earth. After deletions, the revised road network was imported back into ArcGIS so that the sidewalk distances could be calculated.

##### Road measures

Four neighborhood road measures (total length of roads, percentage of roads with speed limits less than or equal to 60 km/h, speed limit of the school’s road, and street connectivity) were obtained from CanMap Route Logistics in ArcGIS software. The CanMap Route Logistics database provides geospatial information on roads and their segments (e.g., blocks) across the country. This includes information on the length of each road segment and its speed limit. Total length of roads was calculated in ArcGIS as the distance of all road segments within the 1 km buffer. The percentage of roads with speed limits ≤60 km/h (37 miles/h) was calculated by dividing the length or all road segments within the buffer with speed limits ≤60 km/h by the total length or roads within the buffer. The speed limit of each school’s road was categorized as ≤40 km/h (25 miles/h), 50 km/h (31 miles/h), or ≥60 km/h based on the speed limit of the road segment at the school’s main entrance. The remaining three variables were obtained for the 1 km radial buffer surrounding the school. Street connectivity was calculated as a composite measure of intersection density, average block length, and connected node ratio, similar to measures identified by others [32-34]. Intersection density was calculated as the number of intersections divided by the total land area in each buffer. Average block length was calculated as the total length of roads divided by the number of intersections. The connected node ratio was calculated by dividing the number of true intersections by the number of all intersections including cul-de-sacs and dead ends. Based upon prior work [34], a principal component factor analysis showed that each street connectivity variable was related; factor loadings were: 0.93, 0.66, 0.89 for intersection density, average block length, and connected node ratio variables respectively (Cronbach’s alpha standardized = 0.78). These variables were combined with equal weight, than ranked as a composite variable.

##### SES

Neighborhood SES was measured in the 1 km buffer surrounding each school, based upon the 2006 Canadian Census, using PCensus for MapPoint software. The overall median household income was calculated by weighting each census block by the total population. This variable was categorized into quartiles.

##### Climate

Annual climate variables (calculated from at least 15 years of data between 1971 and 2000) were obtained for each school using the Environment Canada National Climate Data as inferred from the most proximal weather station to each school. These measures included: average temperature (°C), average annual rainfall (mm), average annual snowfall (cm), average annual number of extreme hot days (maximum temperature >30°C or >80°F), and average number of extreme cold days (minimum temperature < −20°C or < −4°F). Each climate variable was categorized into quartiles.

### Statistical analysis

Statistical analyses were performed with SAS version 9.2 (SAS Inc., Carry, NC). Potential correlates of active transportation were initially described for the sample using conventional descriptive statistics. They were further described by the percentage of individuals in each category that engaged in active transportation to school.

Prior to performing multi-level analyses, an empty model was run to calculate the intra-class correlation (ICC), which reflects the proportion of the total variance in the active transportation outcome explained by the area-level. An ICC value of 18% was obtained, suggesting that a large amount of variation was accounted for by school and neighborhood characteristics [35]. This justified the use of multi-level analytical techniques in subsequent analyses.

Our approach to statistical modeling was governed by the following strategy. Due to the exploratory nature of our investigation, a backwards selection approach was employed. Studies of the built environment and walking and bicycling have found more variation at the individual- vs. area-levels [36,37]. Therefore, we performed multi-level modeling in steps, beginning with building a model at the individual-level (individual and family), followed by the introduction of variables at the area-level (school and neighborhood). Our goal was to create a parsimonious list of potential correlates of active transportation to school while controlling for all of the selected variables at multiple levels.

Our multi-level models were then built using the following hierarchal approach: 1) all individual-level variables were considered in bivariate models with active transportation to school as the outcome (multivariate model 1); 2) backwards selection methods were performed next, with a cut-off value of p < 0.05 for retention of individual-level variables; 3) area-level variables were then added to the significant individual-level variables from multivariate model 1 to create multivariate model 2; 4) backwards selection was performed for the area-level variables in multilevel model 2, this time with a cut-off value of p < 0.20 as power was less for the area-level variables, to create the final model (multivariate model 3). If any variable or dummy variable had significance at p < 0.05 (p < 0.20 for the area level variables), or the test for linear trend across categories was significant (p < 0.05), they were retained in the backwards selection modeling approach. Because the analyses were exploratory, we selected a p < 0.20 for the area-level variables. This was done to ensure that we did not exclude area-level variables in the model building process that could achieve significance, but only after controlling for other covariates.

All models were fit as generalized linear models and were built with the SAS GLIMMMIX procedure with a binomial distribution and a logit link to account for the clustered nature of the data. We assumed random intercepts but fixed effects. A Newton–Raphson with ridging technique was applied to all multilevel logistic models to optimize convergence. Odds ratios were converted to relative risks (RR), as per existing precedents [38], RR = OR / [ (1 – Po) + (OR x Po) ], where Po is the prevalence of active transportation in the referent group for each variable.

Additionally, we calculated the population attributable risk (PAR) to estimate the proportion of active transportation to school attributed to the correlates at the different levels. PAR was calculated based upon the RR estimates generated in multivariate model 3 with the following equation: (Pe(RR-1)) / (1 + Pe(RR-1)) where Pe is the proportion of individuals exposed in similar populations [39]. For variables with more than two categories, PAR was calculated for each of the non-referent categories and then summed to obtain an overall PAR estimate. For variables with an RR less than 1, the effect was inverted to obtain an RR > 1 prior to calculation of the PAR.

## Results

Individual and family characteristics (individual-level) of the urban sample of students who lived in close proximity of their school are profiled in Table 1. A total of 3 997 students were available for analysis, with approximately equal numbers of boys and girls. The majority of the students were in grades 6 to 8, and there was considerable variation in social factors relevant to Canadian families and students’ possible choices to engage in active transportation to school. With respect to the study outcome, 62.6% engaged in regular active transportation to school. Table 2 further describes the distribution of the student sample by school and neighborhood characteristics (area-level) that could potentially impact active transportation choices.

**Table 1:** Individual-level (individual and family) characteristics of urban youth (n = 3 997) sampled for study of active transportation in Canada

**Characteristic**	**N (%)**
Gender	
Male	1 930 (48.3)
Female	2 067 (51.7)
Grade	
6	918 (22.8)
7	915 (22.9)
8	932 (23.3)
9	599 (15.0)
10	639 (16.0)
Ethnicity	
Caucasian only	2 443 (61.1)
Caucasian and other	201 (5.0)
Aboriginal	396 (9.9)
Other	957 (23.9)
Number of siblings	
0	608 (15.2)
1	1 729 (43.3)
2+	1 660 (41.5)
Adults at home	
Both mother and father	2 655 (66.4)
One parent and one step-parent	418 (10.5)
Single parent	775 (19.4)
Other	149 (3.7)
Family SES	
Very well off	904 (22.6)
Well off	1 317 (32.9)
Average	1 405 (35.2)
Not very well off	266 (6.7)
Not at all well off	105 (2.6)
Number of cars in household	
0	176 (4.4)
1	1 186 (29.7)
2+	2 635 (65.9)
Residential neighborhood is safe for children	
Strongly agree	1 109 (27.7)
Agree	1 792 (44.8)
Neither agree nor disagree	750 (18.8)
Disagree	242 (6.1)
Strongly disagree	104 (2.6)

**Table 2:** Area-level characteristics of the schools and neighborhoods of urban Canadian youth (n = 3 997)

	**N (%)**	**Median (IQR)**
**School Characteristics**		
Bicycle storage available in a safe location		
No	835 (20.9)	
Yes	3 162 (79.1)	
Has walk/bike to school days and/or walking school bus programs		
No	2 751 (68.8)	
Yes	1 246 (31.2)	
Identification of safe walking/biking routes to school		
No	2 347 (58.7)	
Yes	1 650 (41.3)	
**Neighborhood Characteristics**		
Sidewalk leading to school		
No	161 (4.0)	
Yes	3 836 (96.0)	
% of roads with sidewalks		64.6 (47.4 - 83.0)
Speed limit of school’s road (km/h)		
≤40	311 (7.8)	
50	3 390 (84.8)	
≥60	296 (7.4)	
% of roads with speed limit ≤60 km/h		
<90	493 (12.3)	
90 – 93.99	851 (21.3)	
94 – 99.99	1 196 (29.9)	
100	1 457 (36.5)	
Total length of roads (km)		36.7 (29.5 – 40.9)
Street connectivity		
1 (lowest connectivity)	1 176 (29.4)	
2	1 034 (25.9)	
3	951 (23.8)	
4 (highest connectivity)	836 (20.9)	
Litter in neighborhood		
No problem	1 285 (32.1)	
Minor problem	2 216 (55.4)	
Moderate problem	399 (10.0)	
Major problem	97 (2.4)	
Vacant or shabby housing		
No problem	2 999 (75.0)	
Minor problem	750 (18.8)	
Moderate problem	192 (4.8)	
Major problem	56 (1.4)	
Neighborhood SES (median family income, $CAD)		70 432 (58 129 – 84 063)
Average temperature (°C)		4.7 (2.5 - 7.6)
Average annual rain (mm)		1 369 (609 – 1 747)
Average annual snow (cm)		231 (195–304)
Average number of hot days		4.5 (0.5 - 11.4)
Average number of cold days		26.0 (0.6 - 47.7)

*IQR* = interquartile range.

Table 3 summarizes bivariate, then adjusted (multivariate model 1), associations between each individual-level variable and engagement in active transportation to school. While the bivariate analyses indicated that a number of individual-level factors are potential correlates of active transportation, multivariate model 1 (individually adjusted) results suggested a more modest list of correlates. Table 4 extends these results through the examination of area-level correlates of the school and neighborhood; few of these factors achieved statistical significance. The final multi-level model (multivariate model 3) is presented in Table 5.

**Table 3:** Bivariate and multivariate (Model 1) relationships of individual-level characteristics and active transportation to school (N = 3 997)

**Individual-level characteristics**	**% Engaged in active transportation**	**Bivariate model**	**Multivariate model 1**
		**RR (95% CI)**	**RR (95% CI)**
Gender			
Male	67.6	1.00	1.00
Female	57.9	**0.86 (0.81 - 0.91)**	**0.85 (0.80 - 0.90)**
Grade			
6	58.6	1.00	1.00
7	62.3	1.06 (0.97-1.15)	1.08 (0.99 - 1.17)
8	64.1	1.07 (0.97-1.17)	1.09 (0.99 - 1.19)
9	61.9	1.12 (0.99-1.23)	1.11 (0.99 - 1.23)
10	67.3	**1.14 (1.01-1.25)**	1.13 (1.00 - 1.24)
P trend		**0.03**	0.05
Ethnicity			
Caucasian only	60.3	1.00	1.00
Caucasian and other	70.2	1.09 (0.95 - 1.21)	1.11 (0.97 - 1.23)
Aboriginal	69.2	1.08 (0.98 - 1.18)	1.03 (0.92 - 1.14)
Other	64.1	0.92 (0.84 - 1.00)	0.92 (0.84 - 1.00)
Number of siblings			
0	65.5	1.00	1.00
1	60.1	**0.92 (0.84 - 1.00)**	0.96 (0.88 - 1.03)
2+	64.2	0.97 (0.90 - 1.05)	1.01 (0.93 - 1.08)
P trend		0.88	0.38
Adults at home			
Both mother and father	59.6	1.00	1.00
One parent and one step-parent	67.5	**1.11 (1.02 - 1.20)**	1.08 (0.98 - 1.17)
Single parent	69.0	**1.13 (1.06 - 1.19)**	1.06 (0.98 - 1.14)
Other	69.1	1.13 (0.97 - 1.26)	1.08 (0.92 - 1.23)
Family SES			
Very well off	57.1	1.00	1.00
Well off	62.3	**1.09 (1.00 - 1.21)**	1.07 (0.99 - 1.15)
Average	66.1	**1.17 (1.10 - 1.25)**	**1.15 (1.07 - 1.23)**
Not very well off	66.2	**1.16 (1.04 - 1.28)**	**1.14 (1.00 - 1.26)**
Not at all well off	59.1	1.02 (0.82 - 1.20)	0.97 (0.77 - 1.16)
P trend		**0.0005**	**0.009**
Number of cars in household			
0	76.7	1.00	1.00
1	68.6	0.94 (0.82 - 1.04)	0.94 (0.82 - 1.03)
2+	59.0	**0.85 (0.72 - 0.95)**	**0.85 (0.73 - 0.97)**
P trend		**<.0001**	**0.0001**
Residential neighborhood is safe for children			
Strongly agree	63.5	1.00	1.00
Agree	57.9	1.02 (0.96 - 1.08)	1.01 (0.94 - 1.07)
Neither agree nor disagree	63.6	0.99 (0.91 - 1.06)	0.96 (0.88 - 1.04)
Disagree	63.0	**0.87 (0.75 - 0.99)**	**0.84 (0.72 - 0.96)**
Strongly disagree	62.2	0.98 (0.81 - 1.14)	0.96 (0.77 - 1.12)
P trend		0.14	**0.036**

*RR (95% CI)* = relative risk (95% confidence interval).

**Table 4:** Bivariate and multivariate (Model 2) relationships of area-level characteristics and active transportation to school (N = 3 997)

**Area-level characteristic**	**% Engaged in active transportation**	**Bivariate model**	**Multivariate model 2**
		**RR (95% CI)**	**RR (95% CI)**
**School Characteristics**			
Bicycle storage available in a safe location			
No	68.3	1.00	1.00
Yes	61.1	*0.89 (0.74 - 1.01)*	0.94 (0.77 - 1.09)
Has walk/bike to school days and/or walking school bus programs			
No	64.3	1.00	1.00
Yes	58.8	*0.90 (0.77 - 1.02)*	0.91 (0.74 - 1.06)
Identification of safe walking/biking routes to school			
No	64.4	1.00	1.00
Yes	60.1	0.93 (0.80 - 1.05)	0.95 (0.79 - 1.09)
**Neighborhood Characteristics**			
Sidewalk leading to school			
No	57.1	1.00	1.00
Yes	62.8	1.11 (0.79 - 1.37)	1.10 (0.74 - 1.39)
% of roads with sidewalks			
1 (1.45 - 47.20)	58.1	1.00	1.00
2 (47.21 - 64.30)	58.3	1.06 (0.87 - 1.23)	1.11 (0.87 - 1.32)
3 (64.31 - 84.49)	64.2	1.12 (0.93 - 1.27)	1.14 (0.88 - 1.35)
4 (84.50 – 100)	70.4	*1.16 (0.97 - 1.32)*	1.03 (0.76 - 1.27)
P trend		*0.071*	0.86
Speed limit of school’s road (km/h)			
≤40	59.5	1.00	1.00
50	62.3	*1.15 (0.92 - 1.34)*	*1.27 (0.90 - 1.50)*
≥60	69.6	*1.23 (0.94 - 1.43)*	*1.32 (0.96 - 1.53)*
P trend		*0.12*	*0.13*
% of roads with speed limit ≤60 km/h			
1 (<90)	60.9	1.00	1.00
2 (90 – 93.99)	65.1	1.10 (0.89 - 1.27)	1.06 (0.82 - 1.27)
3 (94 – 99.99)	60.1	1.06 (0.85 - 1.24)	1.08 (0.84 - 1.29)
4 (100)	63.8	1.03 (0.83 - 1.21)	1.05 (0.81 - 1.25)
P trend		0.94	0.78
Total length of roads (km)			
1 (10.7 – 29.2)	55.6	1.00	1.00
2 (29.3 – 37.0)	60.8	1.10 (0.92 - 1.27)	0.99 (0.72 - 1.25)
3 (37.1 – 41.7)	60.8	*1.16 (0.97 - 1.33)*	1.10 (0.79 - 1.37)
4 (41.71 – 73.7)	73.7	**1.32 (1.15 - 1.46)**	*1.27 (0.94 - 1.52)*
P trend		**0.0005**	*0.070*
Street connectivity			
1 (lowest connectivity)	55.8	1.00	1.00
2	66.0	**1.20 (1.02 - 1.36)**	1.07 (0.81 - 1.30)
3	60.7	*1.16 (0.97 - 1.32)*	0.96 (0.65 - 1.25)
4 (highest connectivity)	70.2	**1.24 (1.06 - 1.39)**	0.91 (0.56 - 1.25)
P trend		**0.020**	0.45
Litter in neighborhood			
No problem	62.1	1.00	1.00
Minor problem	61.6	1.03 (0.89 - 1.15)	1.05 (0.88 - 1.19)
Moderate problem	62.9	0.99 (0.77 - 1.18)	1.11 (0.82 - 1.33)
Major problem	91.8	**1.48 (1.24 - 1.57)**	*1.48 (1.17 - 1.58)*
P trend		*0.13*	*0.055*
Vacant or shabby housing			
No problem	62.7	1.00	1.00
Minor problem	59.7	0.98 (0.81 - 1.13)	0.91 (0.71 - 1.10)
Moderate problem	72.4	1.07 (0.81 - 1.28)	*0.76 (0.44 - 1.09)*
Major problem	60.7	1.07 (0.59 - 1.40)	0.73 (0.23 - 1.29)
P trend		0.70	*0.11*
Neighborhood SES (median family income, $CAD)			
1 (32 984 – 56 979)	66.5	1.00	1.00
2 (56 980 – 67 400)	66.1	1.01 (0.85 - 1.15)	0.97 (0.75 - 1.16)
3 (67 400 – 80 300)	60.7	0.94 (0.77 - 1.10)	1.02 (0.80 - 1.19)
4 (80 301 – 108 010)	58.9	*0.88 (0.72 - 1.04)*	0.95 (0.69 - 1.16)
P trend		*0.086*	0.77
Average temperature (°C)			
1 (−4.45 – 2.75)	62.4	1.00	1.00
2 (2.76 – 4.40)	58.9	1.00 (0.82 - 1.16)	*0.71 (0.34 - 1.13)*
3 (4.41 – 7.40)	59.5	1.00 (0.83 - 1.16)	0.79 (0.30 - 1.30)
4 (7.41 – 10.60)	68.3	1.04 (0.87 - 1.19)	0.88 (0.27 - 1.42)
P trend		0.64	0.95
Average annual rain (mm)			
1 (326 – 615)	61.7	1.00	1.00
2 (616 – 1335)	62.1	1.02 (0.84 - 1.18)	0.93 (0.59 - 1.23)
3 (1336 – 1700)	66.3	1.01 (0.84 - 1.16)	1.25 (0.79 - 1.50)
4 (1701 – 3360)	60.6	1.02 (0.85 - 1.18)	1.27 (0.82 - 1.50)
P trend		0.82	0.33
Average annual snow (cm)			
1 (87 – 200)	68.2	1.00	1.00
2 (201 – 240)	70.0	1.04 (0.89 - 1.16)	1.10 (0.87 - 1.26)
3 (241 – 310)	51.7	**0.85 (0.69 - 0.99)**	0.88 (0.59 - 1.13)
4 (311 – 690)	61.8	0.94 (0.79 - 1.07)	0.84 (0.46 - 1.17)
P trend		*0.11*	*0.19*
Average annual number of hot days			
1 (0 – 0.63)	56.4	1.00	1.00
2 (0.64 – 4.65)	63.5	*1.13 (0.94 - 1.30)*	1.19 (0.84 - 1.46)
3 (4.66 – 10.50)	67.8	1.12 (0.92 - 1.28)	1.07 (0.70 - 1.39)
4 (10.51 – 26.00)	65.4	*1.14 (0.95 - 1.31)*	1.15 (0.74 - 1.46)
P trend		*0.16*	0.69
Average annual number of cold days			
1 (0 – 4.5)	63.3	1.00	1.00
2 (4.6 – 26.0)	60.9	0.99 (0.82 - 1.14)	1.17 (0.75 - 1.42)
3 (26.1 – 50)	64.9	1.03 (0.86 - 1.18)	1.17 (0.62 - 1.46)
4 (50.1 – 110.5)	61.5	0.96 (0.78 - 1.13)	1.14 (0.45 - 1.49)
P trend		0.87	0.69

Model 2 controls for the significant variables by backwards selection from the first model (gender, family structure, family SES, number of cars in household, and perceived residential neighborhood safety).

*RR (95% CI)* = relative risk (95% confidence interval).

**Table 5:** Final multivariate model of the relationships of characteristics of the individual and family, school, and neighborhood with active transportation to school (N = 3 997)

**Characteristic**	**Multivariate model 3**
	**RR (95% CI)**
**Individual and Family Characteristics (Individual-level)**	
Gender	
Male	1.00
Female	**0.86 (0.80 - 0.91)**
Family structure, living with:	
Both mother and father	1.00
One parent and one step-parent	**1.10 (1.00 - 1.19)**
Single parent	1.07 (0.99 - 1.14)
Other	1.10 (0.94 - 1.24)
Family SES	
Very well off	1.00
Well off	1.08 (1.00 - 1.16)
Average	**1.16 (1.08 - 1.24)**
Not very well off	**1.14 (1.01 - 1.26)**
Not at all well off	1.00 (0.80 - 1.19)
P trend	**0.0041**
Number of cars in household	
0	1.00
1	0.94 (0.82 - 1.04)
2+	**0.87 (0.74 - 0.97)**
P trend	**0.0003**
Residential neighborhood is safe for children	
Strongly agree	1.00
Agree	1.00 (0.94 - 1.07)
Neither agree nor disagree	0.95 (0.86 - 1.03)
Disagree	**0.83 (0.70 - 0.95)**
Strongly disagree	0.95 (0.76 - 1.11)
P trend	**0.019**
**School Characteristics (Area-Level)**	
Has walk/bike to school days and/or walking school bus programs	
No	1.00
Yes	*0.89 (0.74 - 1.03)*
**Neighborhood Characteristics (Area-Level)**	
% of roads with sidewalks	
1 (1.45 - 47.20)	1.00
2 (47.21 - 64.30)	1.11 (0.90 - 1.30)
3 (64.31 - 84.49)	*1.17 (0.96 - 1.34)*
4 (84.50 – 100)	1.09 (0.87 - 1.28)
P trend	0.35
Speed limit of school’s road (km/h)	
≤40	1.00
50	*1.22 (0.92 - 1.43)*
≥60	*1.26 (0.96 - 1.47)*
P trend	*0.13*
Total length of roads (km)	
1 (10.7 – 29.2)	1.00
2 (29.3 – 37.0)	1.00 (0.76 - 1.22)
3 (37.1 – 41.7)	1.08 (0.85 - 1.29)
4 (41.71 – 73.7)	*1.23 (1.00 - 1.42)*
P trend	**0.031**
Litter in neighborhood	
No problem	1.00
Minor problem	1.05 (0.89 - 1.18)
Moderate problem	1.09 (0.83 - 1.29)
Major problem	**1.47 (1.16 - 1.57)**
P trend	*0.061*
Vacant or shabby housing	
No problem	1.00
Minor problem	0.95 (0.77 - 1.11)
Moderate problem	0.83 (0.52 - 1.13)
Major problem	0.76 (0.29 - 1.26)
P trend	*0.19*
Average temperature (°C)	
1 (−4.45 – 2.75)	1.00
2 (2.76 – 4.40)	*0.77 (0.54 - 1.00)*
3 (4.41 – 7.40)	*0.76 (0.46 - 1.08)*
4 (7.41 – 10.60)	0.87 (0.53 - 1.19)
P trend	0.71
Average annual rain (mm)	
1 (326 – 615)	1.00
2 (616 – 1335)	0.94 (0.68 - 1.18)
3 (1336 – 1700)	1.16 (0.76 - 1.42)
4 (1701 – 3360)	*1.25 (0.91 - 1.45)*
P trend	0.20
Average number of hot days	
1 (0 – 0.63)	1.00
2 (0.64 – 4.65)	*1.24 (0.95 - 1.46)*
3 (4.66 – 10.50)	1.18 (0.90 - 1.41)
4 (10.51 – 26.00)	**1.33 (1.05 - 1.53)**
P trend	*0.057*

*RR (95% CI)* = relative risk (95% confidence interval).

The final multivariate model indicates that factors at both individual and area-levels were associated with active transportation. *Individual and family characteristics (individual-level)* included: gender (female: RR = 0.86, 95% CI: 0.80-0.91); living with one parent and one step-parent (RR = 1.10, 95% CI: 1.00-1.19); “well off” (RR = 1.08, 95% CI: 1.00-1.16) to “not very well off” (RR = 1.14, 95% CI: 1.01-1.26) perceived family affluence; two or more cars in the household (RR = 0.87, 95% CI: 0.74-0.97); and “disagree” that the residential neighborhood is safe for children (RR = 0.83, 95% CI: 0.70-0.95). S*chool characteristics (area-level)* included*:* presence of a walk/bike to school day program or a walking school bus program (RR = 0.89, 95% CI: 0.74-1.03). N*eighborhood characteristics (area-level)* included*:* a higher percentage of roads with sidewalks (quartile 3: RR = 1.17, 95% CI: 0.96-1.34); a higher speed limit of the school’s road (50 km/h: RR = 1.22, 95% CI: 0.92-1.43; ≥60 km/h: RR = 1.26, 95% CI: 0.96-1.47); increased total length of roads (quartile 4: RR = 1.23, 95% CI: 1.00-1.42,); litter in neighborhoods perceived as a major problem (RR = 1.47, 95% CI: 1.16-1.57); presence of vacant or shabby housing (p_*trend*_ = 0.19); a lower average daily temperature (quartile 2: RR = 0.77, 95% CI: 0.54-1.00; quartile 3: RR = 0.76, 95% CI: 0.46-1.08); higher amounts of rain (quartile 4: RR = 1.25, 95% CI: 0.91-1.45); and more extreme hot days in a year (quartile 4: RR = 1.33, 95% CI: 1.05-1.53).

Table 6 displays the PAR estimates for the correlates of active transportation to school that were present in the final model (multivariate model 3). We did not calculate the PAR for three variables, including the lack of walk/bike to school days, the presence of litter in the neighborhood, and the speed limit of the school’s street due to the confusing RRs. These variables were ignored, as it does not make sense to reduce active transportation programs in schools, or to increase litter in neighborhoods, or to increase the speed limit of the school’s road in order to increase active transportation to school. PAR estimates ranged from 2.3% to 10.8% (10.8% being the number of cars in the household) for the individual-level variables and from 6.9% to 16.6% (16.6% being average temperature) for the area-level variables. The ratings under potential to intervene column in Table 6 were based upon our judgment on the feasibility of intervening upon each of the listed correlates.

**Table 6:** Population attributable risk and the potential for intervention of the correlates of active transportation to school

**Characteristic**	**PAR**	**Potential to intervene**	**How to intervene**
**Individual Characteristics**			
Female gender	7.1%	High	Safe walking programs directed towards females
Not living with both parents	2.8%	Low	
Low family SES (< very well off)	8.8%	Low	
Cars in household (1 or more)	10.8%	Low	
Low perceived neighborhood safety	2.3%	High	Determine what makes a neighborhood feel safe and direct intervention towards these factors
**Neighborhood Characteristics**			
% of roads with sidewalks (> quartile 1)	9.5%	High	Construction of sidewalks on roads that have none
Total length of roads (> quartile 1)	6.9%	High	Building new schools in areas with more streets; or increasing multi-use trails
No problem with vacant or shabby housing	10.4%	Low	Improve the aesthetics of neighborhoods where children live
Low average temperature (quartile 1)	16.6%	Low	
High total rain (quartiles 3 and 4)	9.8%	Low	
High number of hot days (> quartile 1)	16.1%	Low	

*RR* = Relative Risk, *PAR* = Population Attributable Risk.

## Discussion

We identified the most important hypothesized correlates of active transportation to school in Canadian youth residing close to their school using indicators of: 1) the strength of associations identified via regression analyses; 2) population attributable risk; and 3) the potential for intervention. The most important finding of this national study was that the choice to engage in active transportation to school was governed by multiple factors at the individual- and area-levels, as opposed to one or more very specific factors.

There are major differences between our study design and methods from those used in previous studies in this field. Our study involved a geographically diverse sample from across the country, but at the same time was limited to urban youth who lived in close proximity to their school (ie, within 1 mile or 1.6 km) and would therefore likely not be eligible for school bussing. We measured multiple active transportation correlates at multiple levels, and because of this, employed multi-level analytical approaches.

Despite differences in study design and methods, many of the individual and family correlates that were identified in our study have been identified in previous studies that examined determinants of active transportation, such as gender [6,9-11], family structure [14,15], and the number of cars in the household [17]. However, unlike previous American studies which found that Hispanic and Black students were more likely to engage in active transportation to school [6,12,13], we did not find any associations for ethnicity. This may be related to the differing ethnic minority compositions in the United States and Canada. We also did not find an association between the number of siblings and active transportation, while several others have identified such an association [8,14,15,40]. Finally, most other studies have found that youth with a lower family SES were more likely to engage in active transportation to school [8,14,15,21]. Our results showed more of a U-shape pattern with SES, wherein students in the middle SES categories were the most likely to engage in active transportation to school.

School correlates of active transportation are not well understood as few studies have investigated these associations. Our main finding for schools was that, of the three potential school correlates examined, only one was associated with active transportation. Specifically, and to our surprise, the presence of active transportation programs (walk/bike to school days and walking school bus programs) was negatively associated with active transportation to school. This may be an artifact of the lack of temporality in our cross-sectional study design. That is, it is possible that schools with lower active transportation rates implemented walk/bike to school days and walking school bus programs in an attempt to address this public health issue.

Several neighborhood factors were also correlated with active transportation to school. Consistent with findings from a recent systematic review, we found no association with street connectivity (a measure of the directness of travel routes), but a positive association with the percentage of streets with sidewalks and the total length of streets [20]. Our finding that climate measures were associated with active transportation to school differs from other studies that were not as geographically diverse [6,23]. We also investigated several neighborhood variables that, to date, have been unstudied. Of these, our findings suggest that there is a relationship between aspects of the environment that are related to safety and aesthetics (e.g., presence of sidewalks, presence of shabby housing, presence of litter).

As discussed above, several individual and area-level variables were correlated with active transportation in our study. In order to supply information for the development of informed policy, we identified the most important correlates based upon the strength of the identified association, its population attributable risk, and the potential for intervention (see Table 6). Using these criteria, the most important correlates of active transportation to school were gender, the perception of residential neighborhood safety, the percentage of roads with sidewalks, and the total length of streets. In order to increase female engagement in active transportation to school, interventions could follow similar existing programs, such as the LEAP program implemented in South Carolina [41]. Additionally, other programs, such as ENACT suggest that resident-led neighborhood programs are effective at increasing active transportation to school by improving perception of neighborhood safety, with safety in numbers [42].

At the area-level, we propose that the type of intervention would vary depending on whether it is an existing school or a newly constructed school. For existing schools, improvements to the existing active transportation infrastructure (e.g., traffic calming strategies, cross-walks, bicycle paths, new sidewalks and traffic diversion efforts), such as done by the Safe Routes to School intervention in California, may improve rates of active transportation to school [19]. Newly constructed schools should be built in active transportation friendly environments.

The main limitations of our study include the following methodological issues. First, this study may have been affected by selection, as after excluding multiple schools and students, our final sample was reduced from 26 078 to 3 997 (many students were removed because they did not reside in an urban core, and because they did not live within ~1 mile of their school). Third, there may be measurement error with the distance from school inclusion, as the geographical center of the postal code area was considered as a proxy for the students’ home location. Due to the fact that these analyses were limited to urban areas, the estimated locations should be relatively precise [28]. Fourth, we only have information on the trip from home to school; differences in mode of transportation may exist between journeys going to and leaving from school [5,9]. Fifth, outside of family structure and number of vehicles in the home, we were not able to examine potential parental correlates, and parents are clearly very involved in transportation decisions for their children. Sixth, due to the cross-sectional nature of the data, temporality between the variables cannot be assured. Finally, although there was sufficient power to study the individual and family variables and active transportation to school, power was more limited for the school and neighborhood variables.

## Conclusion

Potential correlates of active transportation (at the individual- and area-levels) among urban youth living within close proximity to their school were examined using multi-level analytical methods. We found that the decision to engage in active transportation to school was affected by multiple factors at multiple levels. We identified gender, perception of residential neighborhood safety, the percentage of streets with sidewalks, and the total length of roads as the most important correlates of active transportation to school. Recommendations for interventions (e.g., safe-walking programs directed towards girls, and improvements to active transportation infrastructure) were made with the purpose of informing the development of future intervention and policy-based research aimed at increasing engagement in active transportation to school in youth.

## Abbreviations

CI: Confidence interval; GIS: Geographic information systems; HBSC: Health Behaviour in School-Aged Children Survey; ICC: Intra-class correlation; OR: Odds ratio; PAR: Population attributable risk; RR: Relative risk; SES: Socioeconomic status.

## Competing interests

The authors declare that they have no competing interests.

## Authors’ contributions

All three authors designed the study. KG completed the statistical analyses with support and advice from WP and IJ. KG wrote the initial draft of the paper, which was extensively edited by WP and IJ. All authors read and approved the final manuscript.
